# Effect of Cross-Linking Degree of EPDM Phase on the Morphology Evolution and Crystallization Behavior of Thermoplastic Vulcanizates Based on Polyamide 6 (PA6)/Ethylene-Propylene-Diene Rubber (EPDM) Blends

**DOI:** 10.3390/polym11091375

**Published:** 2019-08-21

**Authors:** Lifeng Ma, Wei Yang, Hui Guo

**Affiliations:** 1 School of Mechanical and Automotive Engineering, Shanghai University of Engineering Science, Shanghai 201620, China; 2 College of Polymer Science and Engineering, State Key Laboratory of Polymer Materials Engineering, Sichuan University, Chengdu 610065, China

**Keywords:** thermoplastic vulcanizates (TPVs), dynamic vulcanization, morphology evolution, crystallization behavior

## Abstract

As a special class of “green” elastomers, thermoplastic vulcanizates (TPVs) have been widely used in industries due to the combination of the excellent resilience of conventional elastomers and the easy recyclability of thermoplastics. Here, the morphology evolution of TPVs based on polyamide 6/ethylene-propylene-diene rubber (PA6/EPDM) blends was investigated by varying the content of the curing agent, phenolic resin (PF). With the incorporation of 6 wt% PF, the gel content of the EPDM phase reaches a high value of 49.6 wt% and a typical sea-island structure is formed with EPDM domain in a micro-nano size. The dynamic rheology behaviors of TPVs showed that with the curing degree of EPDM phase increasing, a denser network of EPDM particles is formed in PA6 matrix. Additionally, a lower crystal degree and crystal peak temperature are observed, indicating that there exists a growth restriction of PA6 crystal plate induced by a thinner plastic layer between the adjacent EPDM particles. However, the crystal form of PA6 is not changed with the increasing curing degree of the EPDM phase. This study provides an effective strategy to realize a new kind of TPVs, which can be easily introduced into industrial applications.

## 1. Introduction

Thermoplastic vulcanizates (TPVs) are a group of high-performance thermoplastic elastomers prepared by the dynamic vulcanization, which consist of a high content of crosslinked rubber as the dispersed phase and a low content of thermoplastics as the continuous phase [1,2,3,4]. These phase structure characteristics endow TPVs with excellent resilience of conventional vulcanized elastomers and good processability of thermoplastics [5,6,7,8]. Therefore, TPVs have attracted considerable attention in the past two decades, and have been widely used in industries such as automotive industry, building, electronics, etc.

As is well-known, the performance of TPVs strongly depends on their final morphology, which are affected by many factors, such as cross-linking degree, composition ratio, viscosity ratio of the two individual components, the processing condition, etc. [9,10,11,12,13]. Machado et al. [11] studied the effect of cross-linking on the phase inversion of polypropylene (PP) and ethylene-propylene-diene rubber (EPDM) blends and found that the position of the phase inversion region is essentially governed by the composition ratio and is independent of the viscosity ratio of PP/EPDM blends. Tian and co-workers [10] studied the relationship of the cross-linking degree and mechanical properties of TPVs during dynamic vulcanization processing and found that a significant improvement in elasticity can be realized with a decreasing rubber particles size of TPVs based on PP/EPDM blends. Therefore, it is much more crucial to achieve high performance TPVs by the means of controlling their final morphology.

Nowadays, TPVs based on PP/EPDM system, a kind of the most widely used TPVs, have experienced a fast development period of technological and academic research. A series of studies have been performed to understand the morphology formation, evolution, and performance of this kind of TPVs [14,15,16]. Radusch et al. [16] have suggested that the initial co-continuous morphology is crucial to achieve the desired fine dispersed EPDM particle, which can effectively transfer the shear and stresses into both the two phases, which is essential to break up the elastomeric phase during dynamic vulcanization. Ning and co-workers [14] found that for TPVs based on PP/EPDM blends the micrometer-sized rubber particles obtained at the end of dynamic vulcanization process are actually agglomerates of EPDM nanoparticles with a diameter of about 40–60 nm.

In recent years, much attention has been devoted to develop new series of TPVs to meet the needs of market requirement and broaden the application of TPVs, such as TPVs based on PA/isobutylene-isoprene rubber (IIR) blends as gas barrier materials [17,18], TPVs based on polyamide (PA)/EPDM blends for high temperature application [19,20], TPVs based on PP/nitrile-butadiene rubber (NBR) and PA/NBR blends as oil-resistance materials [21,22,23], TPV nanocomposites to improve their mechanical strength and dimensional stability or functionalized TPVs [6,24,25,26], etc..

In our previous study, we introduced conductive nanoparticles into TPVs based on PP/EPDM blends and achieved sensing TPV materials actuated by temperature and strain changes [6,24,27,28]. However, to date, much attention still has been paid to the morphology evolution and functionalization of TPVs based on PP/EPDM blends. To broaden the application of TPVs and enhance the overall performance of TPVs, polyamide 6 (PA6) with a better performance has been chosen as the thermoplastic matrix in this study and we aimed to elucidate the effect of cross-linking degree of EPDM phase on the morphology evolution and crystallization behavior of TPVs based on PA6/EPDM blends.

## 2. Materials and Methods

### 2.1. Materials and Sample Preparation

A commercial PA6 (Akulon F136-C, with a melt viscosity of 547.1 Pa·s and a density of 1.13 g/cm^3^) was purchased from DSM Co., Ltd., Heerlen, The Netherlands. The EPDM (Nordel IP 4725P, with 4.9% Ethylidene norbornene (ENB)) was produced by Dow Elastomers Co., Ltd., Wilmington, DE, USA. Phenolic resin (PF), used as the curing agent, was obtained from Yuantai biochemistry industry company, Shanghai, China.

To minimize the effects of moisture, PA6 was firstly dried in a vacuum oven for 24 h at 80 °C prior to the melt blending. The melt-reactive blending process for preparing TPV samples (PA6:EDPM = 50:50 wt%) was carried out in an XSS-300 torque rheometer (Shanghai Kechuang Rubber Plastics Machinery Set Ltd., Shanghai, China) with a rotor speed of 30 rpm and a set temperature of 250 °C. PA6 and EPDM were first added in the mixer, and after 2.5 min, PF (1 wt%, 2 wt%, 4 wt%, and 6 wt% to the total weight of the blends) was added and melt-reactive blending was continued for another 4 min. At last, the mixture was taken out and cut up. For the sake of brevity, TPVs with different PF loading were designated as TPV1 (1 wt%), TPV2 (2 wt%), TPV4 (4 wt%) and TPV6 (6 wt%), respectively. Additionally, PA6/EPDM blends without PF loading were named as TPV0 were prepared with the same procedure and processing parameters for comparison. The mixtures were compression-molded into sheets with a thickness of about 1.0 mm at 240 °C for 10 min under a pressure of 10 MPa.

### 2.2. Characterization

#### 2.2.1. Gel Content

Firstly, 0.5 g of the TPV sample was selectively extracted by formic acid and subsequently the residual sample was dried and packaged in a 120-mesh stainless steel pouch in boiling xylene to dissolve the un-vulcanized EPDM phase to further characterize the gel content.

#### 2.2.2. FTIR Analysis

Several grams of pure EPDM and the residue of TPV samples after Soxhlet extracting were compression molded into thin films between aluminum sheets on a laboratory hot press at 240 °C under 10 MPa. FTIR spectra were determined on a Nicolet 6700 FTIR spectrometer (Nicolet Instrument Company, Waltham, MA, USA) and FTIR-attenuated total reflection spectra were recorded from 650 to 4000 cm^−1^ by averaging 32 scans at a resolution of 2 cm^−1^.

#### 2.2.3. Morphological Observation

The phase morphology of TPV samples was characterized with scanning electron microscope (SEM, JEOL JSM-5900LV, Tokyo, Japan) at an accelerating voltage of 20 kV. Before observation, the fractured surfaces of compression-molded samples were etched with formic acid to remove PA6 and sputtered with gold to avoid the charge accumulation.

#### 2.2.4. Rheological Characterization

The rheological measurements were performed using an AR2000ex stress-controlled dynamic rheometer (TA Corporation, New Castle, DE, USA) with the parallel-plate geometry in a diameter of 25mm. Frequency sweep was carried out in a frequency range of 0.01–100 Hz at 240 °C. The strain used was 2%, which is within the linear viscoelastic range.

#### 2.2.5. Differential Scanning Calorimetry (DSC)

The crystallization and melting behaviors of TPV samples were measured using a differential scanning calorimeter (DSC, TA Q20, New Castle, DE, USA). Then, 3–5 mg samples were heated up to 250 °C rapidly under a nitrogen atmosphere and held at 250 °C for 5 min to eliminate the thermal history. Afterwards, the samples were cooled to 40 °C at a rate of 10 °C/min to record the crystallization behavior, and then heated again to 250 °C at a heating rate of 10 °C/min to record the melting behavior.

#### 2.2.6. Wide-Angle X-ray Diffraction (WAXD)

WAXD measurements were carried out with a DX1000 X-Ray diffractometer (Billerica, MA, USA) at room temperature. The Cu Kα (wave length = 0.154056 nm) irradiation source was operated at 50 kV and 30 mA. Patterns were recorded by monitoring diffractions from 5° to 35°, and the scanning speed was 3°/min.

## 3. Results and Discussion

### 3.1. Dynamic Vulcanization

**Figure 1:**
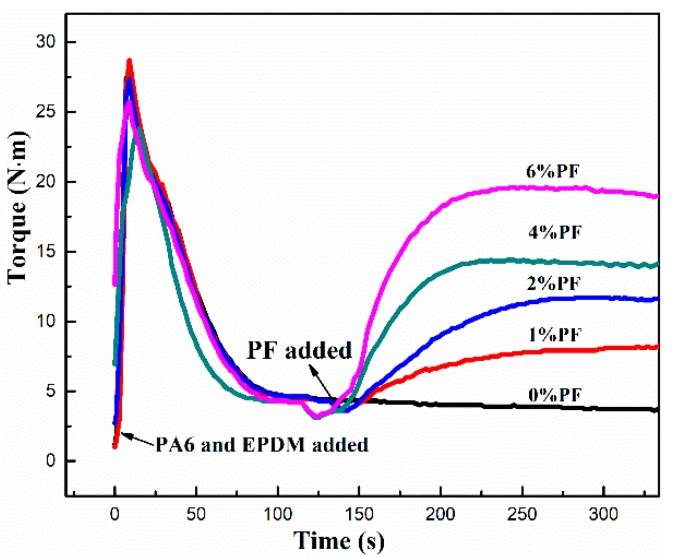
Plot of torque versus time during dynamic vulcanization of thermoplastic vulcanizates (TPVs) with different phenolic resin (PF) loading.

The cross-linking reaction of rubber phase plays a crucial role in the evolution of the morphological structure of TPVs and their final microstructure. Firstly, the variation of torque value as a function of mixing time was recorded and shown in Figure 1. It can be observed that at the initial stage, the torque value increases rapidly with PA6 and EPDM added, and subsequently decreases to a stable value because of the complete melting of the blends. Afterwards, PF resin was added, and accompanied by the rapid vulcanization of EPDM phase, the torque value rises dramatically until it reaches a maximum, then it declines slowly until the end of dynamic vulcanization. Further detailed observation shows that, with PF loading increasing from 1 to 6 wt%, the torque value at the plateau climbs gradually, indicating that the melt viscosity of TPV system is increasing with the increasing PF incorporation. Moreover, the blending time corresponding to the maximum torque value is decreasing with the PF loading increasing. Thus, it can be concluded that the cross-linking rate and efficiency of TPVs are enhanced largely caused by the increasing PF loading.

**Figure 2:**
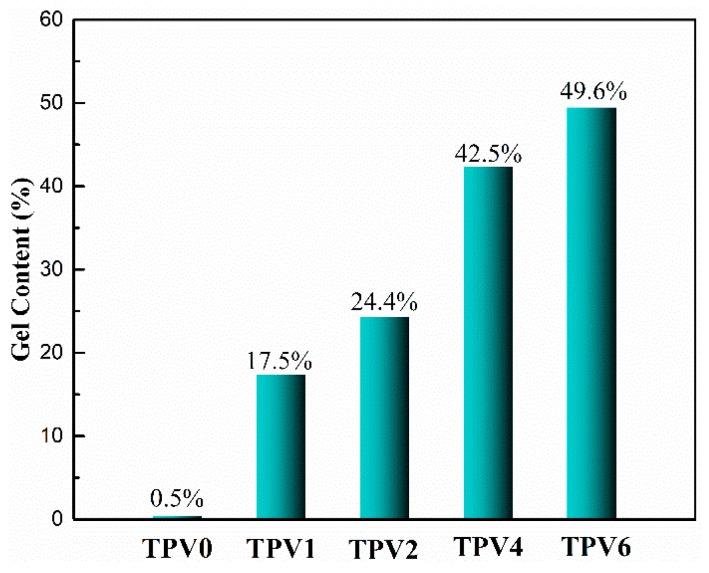
Gel content of TPVs with different PF incorporation.

The residual TPV samples after extraction using Soxhlet extractor were used to further characterize the gel content and to elucidate the cross-linking degree of EPDM phase quantify, as shown in Figure 2. It can be seen that the gel content for TPV0 is only 0.5 wt%, while for TPV samples, with the PF content increasing, the gel content sharply increases to 17.5 wt% for TPV1, and then gradually increases to 49.6 wt% for TPV6. Obviously, EPDM phase can be effectively cured with the addition of PF resin and with increasing PF loading, the gel content increases largely, indicating that the cross-linking degree of EPDM phase increases with the loading of PF resin increasing.

**Figure 3:**
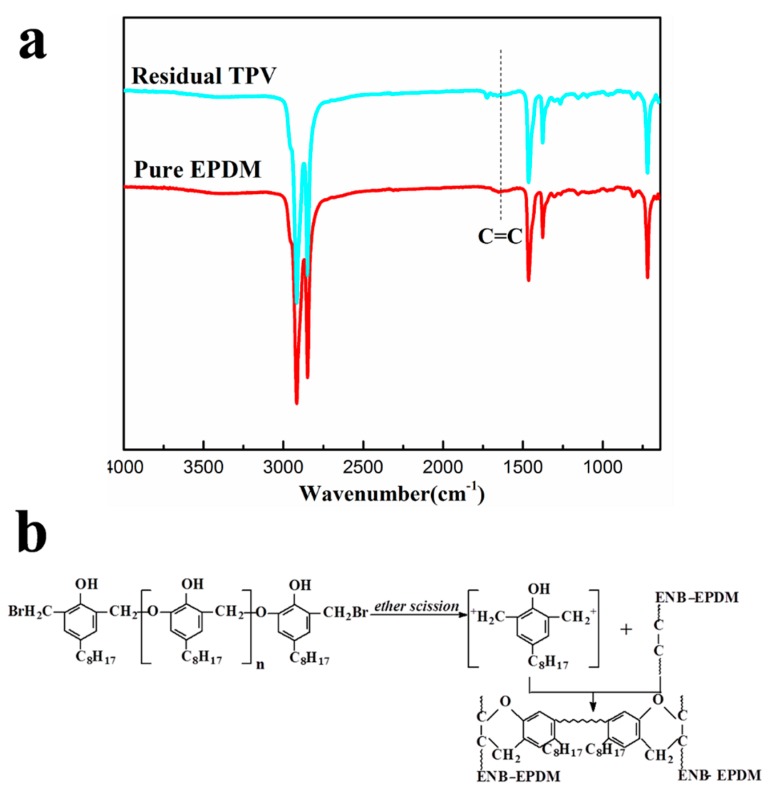
(**a**) FTIR spectra of ethylene-propylene-diene rubber (EPDM) and residual TPV sample after extraction using xylene steam in a Soxhlet extractor; (**b**) a possible schematic drawing of the reactions of PF and EPDM.

Subsequently, the residual samples of TPV6 after extraction were used for FTIR analysis. The FTIR spectra of pure EPDM and TPV samples after extraction using xylene steam in Soxhlet extractor for 48 h are shown in Figure 3a. The characteristic absorption bands at 1700–1550 cm^−1^ are assigned to the C=C bond of diene monomer (ENB) for EPDM. For pure EPDM, the absorption bands at 1650 cm^−1^ assigned to C=C bond appears in the form of a tiny broad peak [29], which should be ascribed to the ultra-low content of ENB (only 4.9 wt%) in EPDM phase. Compared with pure EPDM, the absorption bands attributed to the C=C bonds for TPV6 sample disappear after PF-induced dynamic vulcanization, indicating that the C=C bonds of EPDM have completely reacted with PF resin to form the cross-linking network of EPDM. It is well known that EPDM with ENB as the diene monomer is more reactive toward resole cross-linking than that with dicyclopentadiene (DCPD), 1,4-hexa-diene (HD), or vinylidene norbornene (VNB). This should be ascribed to: (1) the selective cross-linking of the unsaturated diene monomer, (2) the thermo-stable cross-linking network, and (3) high cross-linking efficiency of the EPDM phase at the processing temperature. In the mixing process, not only in the extrusion process, but also in the internal mixer, the PF resin degrades into benzyl cations followed by the diastasis of bromide ion, which eventually reacts with the unsaturated bonds of EPDM and connects EPDM chains via chroman or methylene-bridged structures. Here a possible schematic drawing of the possible reactions of PF and EPDM is given in Figure 3b.

### 3.2. Morphology Evolution

**Figure 4:**
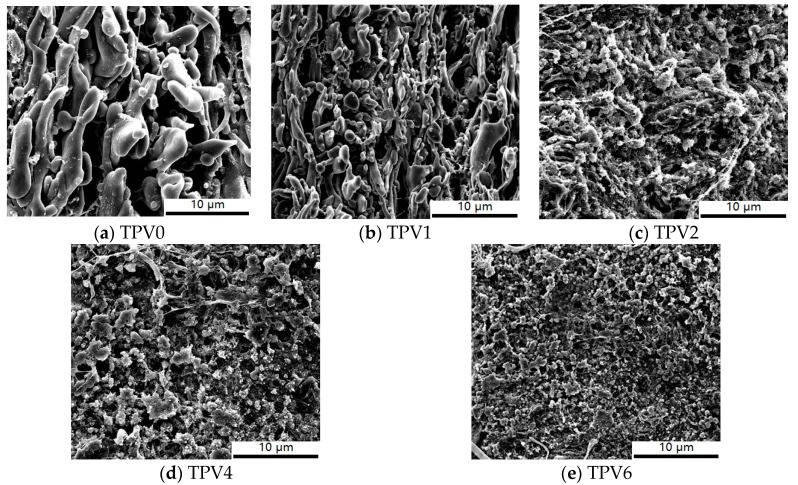
Typical TEM images of TPV0, TPV1, TPV2, TPV4 and TPV6 samples.

**Figure 5:**
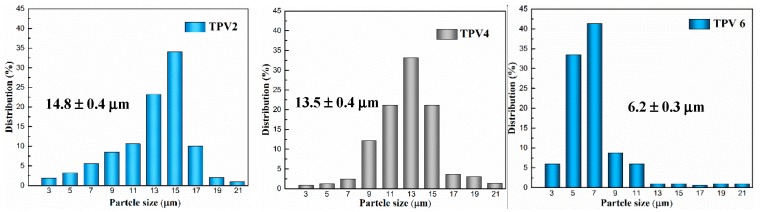
Particle size distribution of TPV2, TPV4 and TPV6 samples.

To explore the morphology evolution of TPVs with different gel content of EPDM, SEM images of these TPVs are shown in Figure 4. To enhance the surface contrast of PA6 and EPDM phase, PA6 has been selectively extracted by formic acid. For TPV0, i.e., PA6/EPDM blends, it can be seen that the approximate double continuous structure has been formed, and with increasing gel content to 17.5% for TPV1, more and more EPDM phase domains have been sheared and broken to a smaller size, which resulted from more efficient transfer of shear force caused by the increasing gel content. The morphology of sample TPV2 is transformed from a co-continuous structure into a sea-island one, indicating that the morphology of TPVs is successfully formed upon PF loading to 2 wt%. Meanwhile, further observation shows that with PF loading increasing further, the phase size of EPDM dispersed phase decreases gradually. To gain a better insight of the detailed microstructure difference of these TPVs, we carried out the quantitative analyses of the average particle diameter and particle size distribution (seen in Figure 5). It can be obtained that the average particle diameter for EPDM phase in TPV2 and TPV4 are about 14.8 ± 0.4 μm and 13.5 ± 0.5 μm, respectively, while that for TPV6 is only 6.2 ± 0.3 μm, indicating that with increasing the cross-linking degree, the average diameter of EPDM phase domains decreases largely.

### 3.3. Rheological Behavior

**Figure 6:**
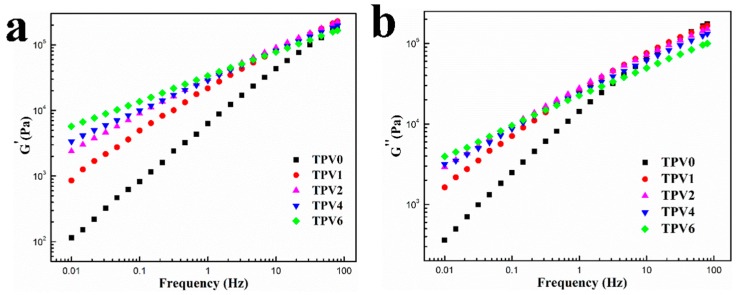
Changes of *G*′(**a**) and *G*″(**b**) as functions of frequency for TPVs with different PF loading.

In our previous work, we used dynamic rheometer to characterize the frequency dependence of tan *δ* of TPV based on PP/EPDM blends, and found that there exists a peak of tan *δ* owing to the EPDM particles, which indicated that the EPDM particles in micro-nano size has formed an effective particle network [30]. In order to further reveal the EPDM particle network evolution of TPVs based on PA6/EPDM blends with different gel content, the oscillatory shear rheological measurements were carried out and the results are shown in Figure 6. Generally, polymer chains can be fully relaxed and a terminal behavior of *G*′∝*ω*^2^ and *G*″∝*ω*^1^ at low frequencies can be observed [31]. As shown in Figure 6a, TPV0 melt exhibits approximately a terminal-like behavior in the low-frequency region. However, this terminal-like behavior becomes invisible when the PF loading is increasing, and the power for *G*′ at low frequencies remarkably decreases with the PF loading increase. The incorporation of PF resin changes the frequency dependence of the dynamic moduli of TPVs. The dependence of *G*′ on *ω* becomes increasingly weaker, indicating that a transition from liquid-like to solid-like viscoelastic behavior occurs [32]. This can be attributed to the formation of a rheological percolation network of EPDM phase when the PF exceeds a critical content, confining the long-range motion of the PA6 chains to a great extent. The detailed data for the slopes, that is, power, *G*′, and *G*″, are listed in Table 1.

As shown in Figure 6b, similar differences in tan δ of the TPVs counterparts can be observed. Lower values of tan *δ* reflect more effective restriction of the EPDM particle networks on the relaxation of PA6 chains, and this restriction effect is directly dependent on the cross-linking degree. In all, the higher *G*’ and lower tan δ at low frequency range for all the counterparts indicate a denser EPDM particle network of TPVs with PF loading increasing. It could be attributed to: (1) a higher elasticity of the network of EPDM phase with the higher curing degree; (2) more compact network of EPDM phase caused by a diameter decrease of EPDM phase with consistent EPDM content as cross-linking degree of EPDM phase increases; and (3) intensified stress superposition between the adjacent rubber particles induced by the much thinner plastic layer with increasing cross-linking of EPDM.

**Figure 7:**
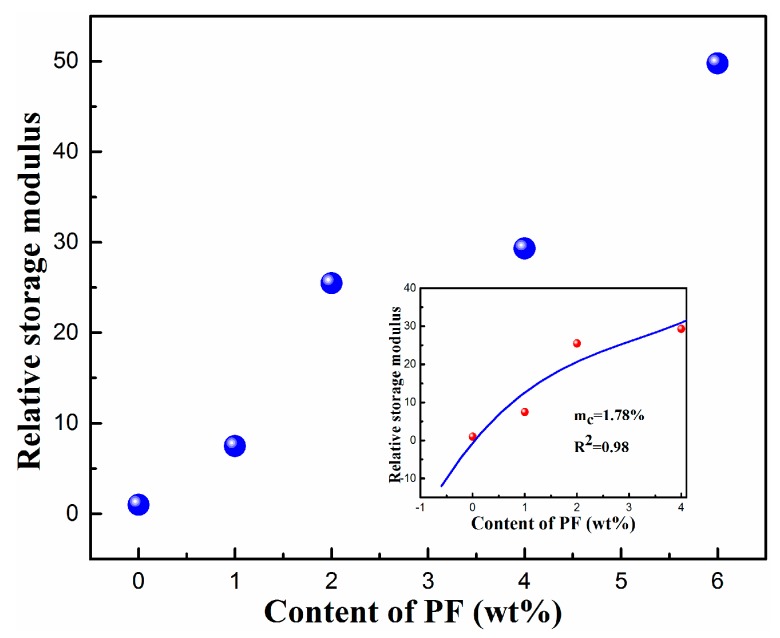
The dependence of relative storage modulus on the content of PF for TPVs.

To make the rheological percolation legible, the dependence of relative storage modulus, $G_{R}^{\prime }\;$, on the content of PF resin is shown in Figure 7. $G_{R}^{\prime }\;$ can be calculated according to Equation (1):

(1) $$G_{R}^{\prime }=G_{TPVX}^{\prime }/G_{PA6/EPDM\;}^{\prime }$$

where $G_{R}^{\prime }\;$ is the relative storage modulus at 0.01Hz, $G_{TPVX}^{\prime }$ is the storage modulus of TPVs with different PF loading, X is the content of PF resin, $G_{PA6/EPDM}^{\prime }$ is the storage modulus of TPV0.

The rheological percolation threshold of TPVs is calculated according to Equation (2):

(2) $$G^{\prime }\propto {\left(m-m_{c,G^{\prime }}\right)}^{\beta_{c,G^{\prime }}}$$

where m is the mass fraction of PF, $m_{c,G^{\prime }}$ is the mass fraction of percolation threshold, and $\beta_{c,G^{\prime }}$ is the critical exponent, which is related to the system dimension.

As shown in Figure 7, the rheological percolation threshold of TPVs is 1.78 wt%, indicating that the EPDM particle network is just formed with a PF content of 1.78 wt%, which is in accordance to the SEM observation. It reveals that the cross-linking degree of EPDM phase affects the EPDM particle network and rheological percolation threshold significantly.

### 3.4. Crystallization Behavior of PA6

**Figure 8:**
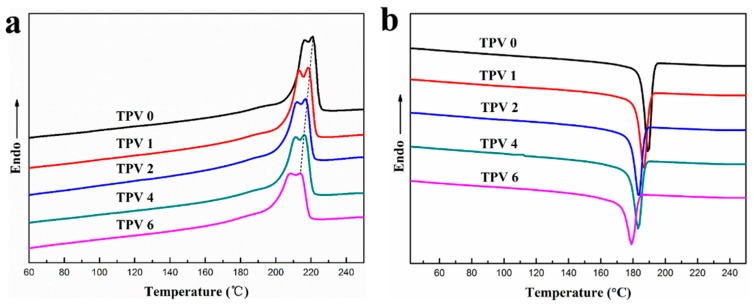
Differential scanning calorimeter scans of TPVs during heating (**a**) and cooling (**b**) for TPVs with different PF loading.

From the above discussion, it is clear that with increasing crosslinking degree of EPDM phase the EPDM phase undergoes a series of changes from the co-continuous structure to the EPDM particle network in PA6 matrix. Moreover, due to the consistent EPDM content, the diameter decrease of EPDM phase would result in a more compact network of EPDM phase and much thinner plastic layer. Therefore, the particle network of EPDM phase notably influences the behaviors of polymer crystallization. Thus, the crystallization and melting behaviors of PA6 matrix in these TPVs with increasing cross-linking degree of EPDM phase are worth to be investigated. Here, the crystallization and melting process of TPVs at a rate of 10 °C min^−1^ were investigated and the results are shown in Figure 8. The peak temperature (*T*_cp_) of non-isothermal crystallization process shifts to a lower temperature visibly with the cross-linking degree of EPDM increasing. It can be seen from Table 2 that the minimum *T*_cp_ is about 10 °C lower than that of PA6/EPDM blends. The heating enthalpy of fusion (Δ*H*_m_) of TPVs is generally lower as the cross-linking degree increases, indicating that the degree of mass crystallinity of PA6 matrix is also decreased, seen in Table 2. This may be due to that the denser EPDM particle network has a greater restriction effect on the mobility of PA6 chains and decreases the crystal growth rate. As for the melting peak temperature, *T*_mp_, TPVs have a lower value than that of PA6/EPDM blend. This observation is probably caused by the fact that a stronger constraint effect for the denser EPDM particle networks goes against the thickening of the crystals and results in a lower *T*_mp_.

The degree of mass crystallinity (*X*_c_) of TPVs was calculated using the following Equation (3) [33]:

(3) $$X_{c}=\frac{∆H_{m}}{∆H_{m}{}^{*}\varphi }\times 100\%$$

where $∆H_{m}$ and $∆H_{m}{}^{*}$ = 240 J/g [34] are melting enthalpy of blends and PA6 with *X_c_* = 100%, respectively; *φ* represents weight fraction of PA6 in TPVs.

It can be seen easily that the crystallinity of TPVs significantly decreases with the incorporation of PF resin, and the maximal decrement of *X*_c_ compared with that of PA6/EPDM blends is about 12.23%. The probable interpretation is that the EPDM particle network affects the crystal growth process of PA6 matrix. For one thing, high cross-linking degree reduces the growth rate of crystals by hindering the motion of PA6 chains due to the denser EPDM particle network; for another, the increase of cross-linking degree decreases the thickness of PA6 matrix between the adjacent EPDM particles, resulting in the growth restriction of PA6 crystal plate.

**Figure 9:**
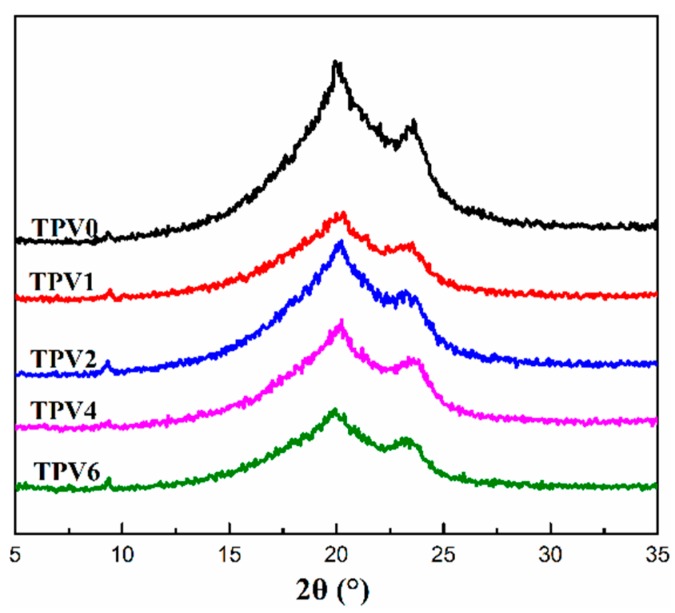
Wide-angle X-ray diffraction curves of TPV series.

To further identify the crystal form of PA6 matrix, the crystalline structure was further characterized by WAXD and the results are shown in Figure 9. It can be seen that there exists two diffraction peaks at 2θ = 20.2° and 23.9° for all the TPV samples, which respectively represent the diffractions of (200) and (002)/(202) planes. It is widely acknowledged that there are two crystal forms of pure PA6, namely α-form and γ-form, corresponding to the diffraction of (200), (002)/(202) planes and (100) plane, respectively [35]. Therefore, the two characteristic diffraction peaks at 2θ = 20.2° and 23.9° for PA6 matrix should be attributed to the diffractions peak of α-form, indicating that there is not the crystal modification transfer of α-form to γ-form.

**Figure 10:**
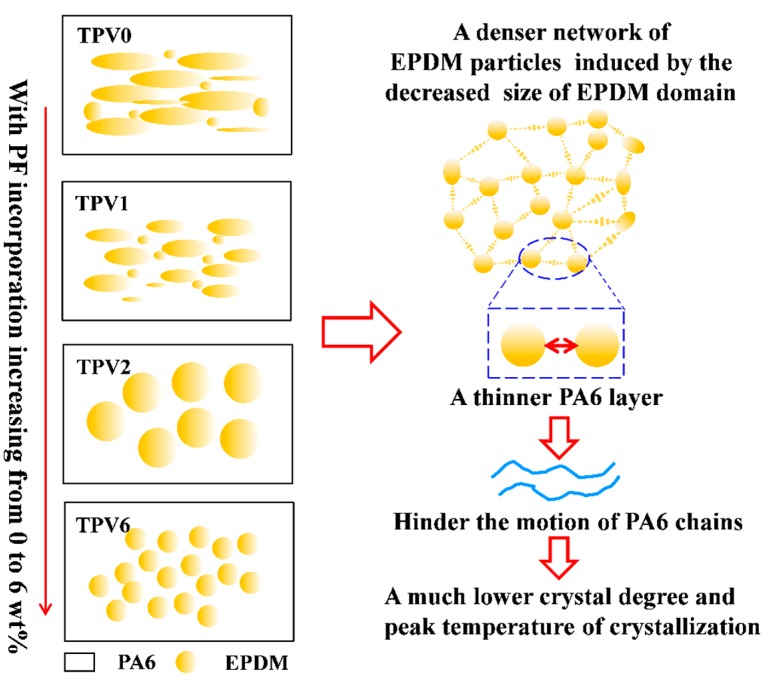
Schematic representation of the structure evolution of TPVs based on PA6/EPDM subjected to an increasing crosslinking degree.

Based on the above analysis, a schematic representation of the phase structure formation for TPVs was proposed and shown in Figure 10. In the initial state, i.e., TPV0 without PF loading, a typical co-continuous structure is formed and as the cross-linking degree increases, the EPDM phase is sheared and broken to a smaller size caused by the efficient transfer of shear. Up to increasing PF loading, the phase inversion finally occurs at a gel content of 24.4%. Subsequently, a denser EPDM particle network is formed with the increased PF incorporation continuously, resulting in incremental hindering effect of the motion of PA6 chains.

## 4. Conclusions

In this study, the effect of cross-linking degree of EPDM phase on the formation of EPDM particle network and crystallization behavior of TPVs based on PA6/EPDM blends were investigated. With the incorporation of PF up to 6 wt%, the gel content of EPDM increases generally to 49.6 wt% and a smaller EPDM particle is obtained with the size of 6.2 ± 0.3 μm. With PF loading increasing, a higher *G*’, and lower tan δ at low frequency have been observed, indicating that there exists a denser EPDM particle network of TPVs caused by the decreased size of EPDM phase, which further gives rise to a growth restriction of PA6 crystal plate, resulting in a much lower crystal degree and peak temperature of crystallization.

## Figures and Tables

**Table 1:** Terminal slopes of *G*′ and *G*″ for TPVs.

Sample	PF (wt%)	*G*′∝*ω*^2^	*G*″∝*ω*^1^
TPV0	0	0.91	0.85
TPV1	1	0.73	0.65
TPV2	2	0.56	0.52
TPV4	4	0.47	0.43
TPV6	6	0.38	0.38

**Table 2:** Differential scanning calorimetry (DSC) parameters of non-isothermal crystallization and the following melting process for TPV series.

Code	*T*_mp_ (℃)	*T*_cp_ (℃)	△*H*m (J/g)	*X*_c_ (%)
TPV0	221.10	189.42	25.64	25.58
TPV1	219.94	185.27	21.29	23.81
TPV2	217.08	183.51	25.51	21.26
TPV4	216.61	183.21	23.62	19.68
TPV6	214.31	179.06	20.89	17.41

*T*_mp_ melting peak temperature, *T*_cp_ crystallization peak temperature, *H*_m_ heat of fusion, and *X*_c_ mass fraction crystallinity.
